# Creating a Caregiver Benefit Finding Scale of Family Caregivers of Stroke Survivors: Development and Psychometric Evaluation

**DOI:** 10.3389/fpsyt.2020.00734

**Published:** 2020-07-29

**Authors:** Yong-xia Mei, Bei-lei Lin, Wei-hong Zhang, Shan-shan Wang, Zhen-xiang Zhang, Dong-bin Yang, Daphne Sze Ki Cheung

**Affiliations:** ^1^School of Nursing and Health, Zhengzhou University, Zhengzhou, China; ^2^Department of Neurosurgery, The People’s Hospital of Hebi, Hebi, China; ^3^School of Nursing, The Hong Kong Polytechnic University, Hong Kong, Hong Kong

**Keywords:** benefit finding, family caregivers, stroke, scale development, validation

## Abstract

In recent years, increased attention has been paid to the benefit finding of family caregivers due to the important role they play. Although some instruments measure benefit finding of caregivers, they do not comprehensively address it in terms specific to the family caregivers of stroke survivors, who require long-term, consistent care. This study is the first effort to develop a comprehensive Caregiver Benefit Finding Scale for the family caregivers of stroke survivors in a Chinese cultural setting. First, 50 items were extracted from a systematic literature review, and a semi-structured interview was conducted with 20 stroke family caregivers to develop the preliminary version of the scale (Version 1). Second, Delphi procedures with 20 experts were used to revise the first version and create Version 2 (37 items). Another six experts were recruited for content validation. Item content validity index (I-CVI) values ranged from 0.83 to 1.00, and the value of the scale CVI was 0.97. Third, 309 family caregivers completed the Version 2 questionnaire and the Chinese version of the Positive Aspects of Caregiving. Two weeks later, 35 family caregivers once again completed the questionnaires. An exploratory factor analysis produced four components (personal growth, health promotion, family growth, and self-sublimation) and 26 items for Version 3 (the cumulative proportion variance was 74.14%). Subsequently, 311 family caregivers completed Version 3. A confirmatory factor analysis confirmed the structure. The goodness of fit index (GFI) = 0.921, adjusted GFI = 0.901, normal fit index = 0.951, incremental fit index = 0.990, comparative FI = 0.990, and the root mean square error of approximation = 0.02 were within the acceptable range. Criterion-related validity was equal to 0.803. The model-based internal consistency index was 0.845 and the values of the Cronbach’α coefficient of the four dimensions were 0.885–0.953. The split-half reliability was 0.92, and the test-retest reliability was 0.994. These findings provide preliminary evidence of the validity and reliability of the Caregiver Benefit Finding Scale. The scale can help researchers and clinicians to achieve a more comprehensive understanding of stroke family caregivers’ positive experience. This understanding is necessary for future efforts to address issues in benefit finding by targeting the underlying mechanism and intervention.

## Introduction

Strokes pose a major health threat and are, on a global scale, the leading cause of mortality and disability (1). In China, stroke is the leading cause of death and the most common origin of diseases that cause disabilities (2). More than 4.5 million stroke survivors live with the resultant disabilities (3) and are consistently being cared for by family caregivers. Caregiving is detrimental to the physical and psychological health of caregivers, a fact that also affects the care recipients’ quality of care and quality of life (4–7).

Caregivers, however, also experience benefits such as personal growth, better relationships with patients, and finding personal meaning during the caregiving experience (8, 9). More importantly, benefit finding may mitigate caregiver burdens, reduce the negative impact on the caregiver’s quality of life, and help caregivers cope with stress caused by caregiving (10, 11). Intervention focused on benefits finding was found to reduce caregivers’ depression and promote caregivers’ physical health by effectively strengthening their immunity (12–14). Hence, in recent years there has been increased attention on measuring the benefit finding for caregivers in the field, to determine benefits finding, explore its effects on caregiver outcomes, and uncover how it works (9, 15).

Some instruments have been developed to measure positive outcomes related to caregiving. These include the Stress Related Growth Scale (16) and Post Traumatic Growth Inventory (PTGI) (17). The validity of these two scales, however, has been questioned (18–21). Other than these particular instruments developed for diverse caregiver populations, some measurements were developed for specific types of caregivers. Positive Aspects of Caregiving (22) was developed for caregivers of dementia patients and the Gains in Alzheimer’s Care Instrument (23) and Reward of Caregiving Scale (24) were developed for palliative caregivers. The Scale for Positive Aspects of Caregiving Experience was developed for caregivers of schizophrenia patients (25). There is a requirement to validate an existing construct within a new population or assess whether adaptation and additions to the construct are required for it to translate to stroke samples.

Benefits finding is the most commonly reported type of meaning-focused coping, which is very important in the revised stress and coping model (26). A meta-analysis defined benefit finding as the positive effects that result from a traumatic event (27). A integrative review of the literature highlighted that benefit finding is an important component of positive outcomes of caregiving (9). Scales have been developed to specifically measure benefit finding, such as the Benefit Finding Scale and Benefit Finding in Multiple Sclerosis Scale. The Benefit Finding Scale was developed for patients with breast cancer (28). This scale has been used with caregivers of patients with other types of cancer (29) and as well as those with heart failure (30). The Benefit Finding in Multiple Sclerosis Scale was developed for caregivers of those with multiple sclerosis (31). These benefit finding scales may not be applicable for caregivers of stroke survivors because the nature of strokes differs significantly from these other diseases.

Moreover, the experience of family caregivers can differ significantly because of specific cultural differences (32). Chinese caregivers may value the benefits gained from receiving praise from the neighborhood, communities, and society in general (33) or from being a role model for younger generations (34). These particular factors are not measured by existing scales. The limitations of the previous scales provided the grounds to conceive the present study to develop a new comprehensive scale, the “Caregiver Benefit Finding Scale for Stroke Caregivers,” and evaluate its psychometric properties.

## Materials and Methods

**Figure 1:**
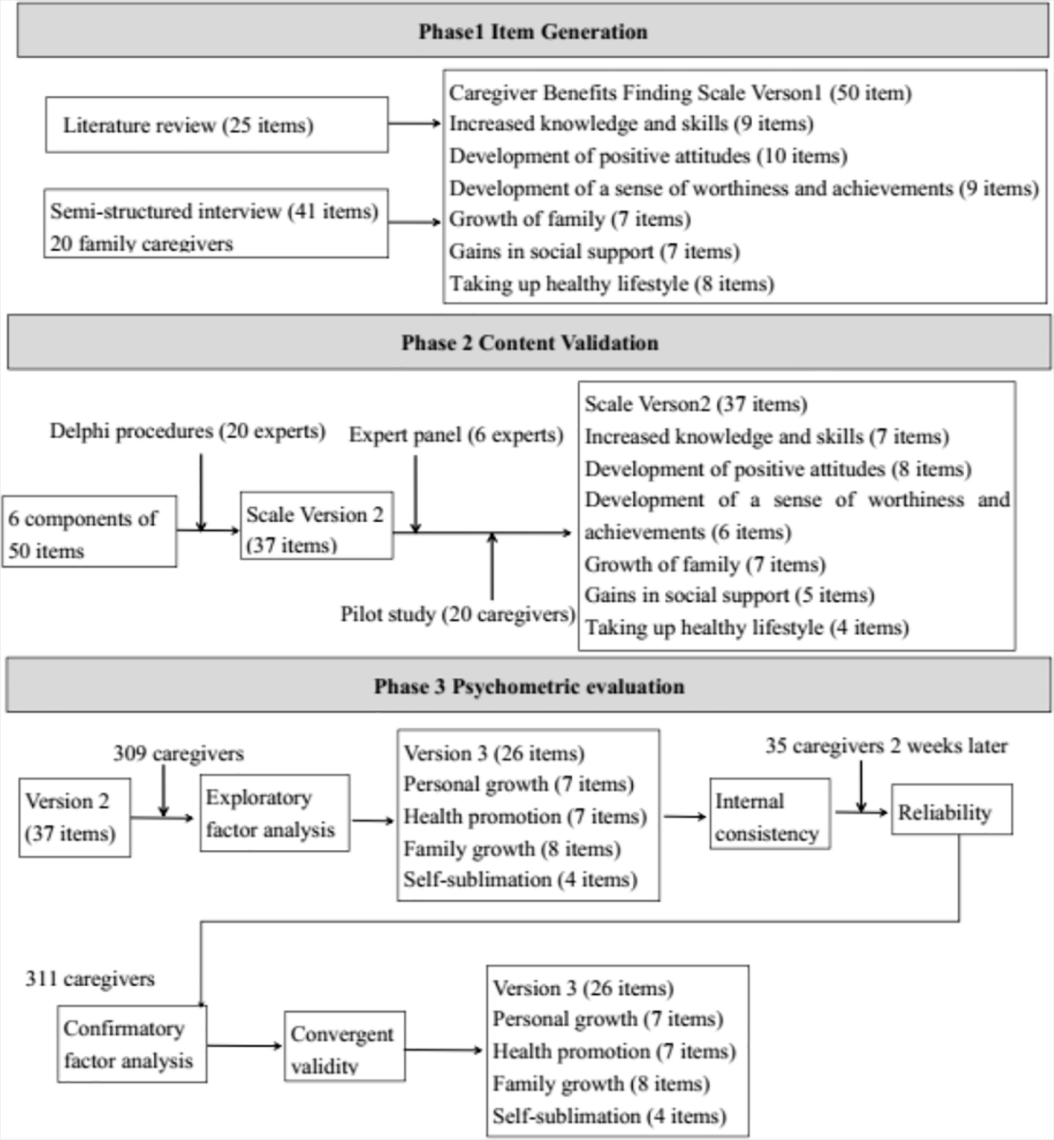
Overview of the three-phased tool validation study.

This study was performed in three phases. In the first phase, a literature review and semi-structured interviews were used to generate items to be included in the questionnaire. In the second phase, Delphi procedures were conducted to revise the item pool and an expert panel was used to assess the content validity. In the third phase, the psychometric evaluation of the Caregiver Benefit Finding Scale was performed. An overview of the tool development process is shown in **Figure 1**.

### Participants and Design

Family caregivers of stroke survivors from five hospitals and four communities in Zhengzhou, China, were recruited to participate in the study. Three inclusion criteria were established for family caregivers. The first criterion was that the caregivers were primary family members (i.e., non-professional and unpaid) of a stroke survivor aged 18 or above, who had a formal diagnosis of cerebrovascular disease and had a functional disability (Barthel Index < 100) (35). The second criterion was that the caregiver provided care for at least 4 h each day for 4 weeks. The third, and final, criterion was that caregiver could communicate in Mandarin and was willing to participate in the study. The study was conducted from June 2015 to December 2017.

### Phase One: Item Generation - Scale Version 1 (50 items)

The Caregiver Benefits Finding Scale, Version 1, was developed through a systematic literature review and semi-structured interviews conducted with family stroke caregivers.

The review was conducted using six English databases (PubMed, CINAHL, PycINFO, Embase, Web of Science, ProQuest Health, and Medical Collection) and three Chinese databases (CNKI, Wanfang, and CMB) to identify previous scales related to caregiving benefits findings. The database search criteria were articles published before December 2015 that used the terms “caregiver” (including synonyms) AND “benefit finding” (including synonyms). The inclusion criteria were articles focused on benefit finding of caregivers. The exclusion criteria were as follows: caregivers were younger than 18 years old; caregivers were professional personnel; patients were younger than 18 years old; and articles not written in English or Chinese. The related existing scales were compared (the details can be found in **Supplementary Table S1** online). All of the items in those scales were read and similar items were removed after discussion with the research team, As a result, 25 items related to benefit finding in family caregivers were obtained.

Semi-structured interviews were performed to determine the perceived benefits from the perspective of the family caregivers and how they interpreted those benefits. The interview included several questions including the following. How have you changed as a result of caring for your wife/husband/father/mother? How have these changes affected your life? What does caring mean to you? What benefits have you perceived that have come to you from being a caregiver? When you have perceived benefits that come from caring, how have your perceptions of the act of caring changed? How do others (other family members, friends, and neighbors)view your caring? What caused you to keep taking care of your wife/husband/father/mother for so long? Purposive sampling and snowball sampling were used to recruit stroke family caregivers. Sample size was determined by the saturation of interview data; the primary researcher perceived that the content of the interviews was becoming repetitive and that no new information was emerging during the interviews, and the interview transcripts were also reviewed by the research team to ensure that no new content was emerging (36). All interviews were performed in the caregivers’ homes and in the presence of two researchers. The interviews were recorded and each interview lasted between 30 and 60 min with an average duration of approximately 42 min. The interviews were transcribed verbatim and then coded independently by two researchers.

Twenty stroke family caregivers were interviewed. Thirteen were females, and seven were male. There were seven wives, five husbands, five daughters, two sons, and one mother of stroke survivors. The caregivers’ duration of care ranged from four months to 14 years. Eight of the caregivers provided care for eight to 12 h each day and six provided care for at least 12 h while six provided care for 4 to 8 h per day. Thematic analysis (including data familiarization, coding and developing themes and subthemes) was performed to analyze the interview transcripts (37). As a result, six themes were identified; increased knowledge and skills, development of positive attitudes, development of a sense of worthiness and achievement, growth of family ties, gains in social support, and adopting a healthy lifestyle. Within the six themes, 41 items were obtained.

As a result of the literature review and semi-structured interviews (and removing 16 duplicate items), a pool of 50 items were generated to measure caregiver benefit finding (for Version 1 of the scale). A five-point Likert scale ranging from 1 to 5 was used to assess the level of benefit finding in caregivers on each item.

### Phase Two: Content Validation - Scale Version 2 (37 items)

The Content of Caregiver Benefit Finding Scale, Version 1, was validated in two rounds of Delphi survey. The 21 national experts were invited by email and 20 responded to the two rounds of Delphi procedures. Of these experts, five were nursing experts in psychology, four were researchers with expertise concerning caregivers of stroke patients, and three were researchers with expertise in scale development. Two were nursing experts working in the community, two were psychiatric nurses, two worked in clinical rehabilitation, two were neurological doctors and one was a nursing expert who worked in stroke clinical wards. In the first round, the initial version of the scale was emailed separately to the experts. A five-point, Likert-type, scale with values ranging from 1 to 5 was used to evaluate the relevance and clarity of the items. The experts could write comments and revise or add items. Discussions were held on the comments by the experts in the research group, and the revision, based on a consensus of opinion, was built. The second round was held 2 weeks later to ensure that there was agreement on the revision. The overall authority grade of the Delphi consultation was 0.895. Kendall’s coefficient of concordance W of the two rounds consultation was 0.138 (χ^2^ = 135.14, *P* < 0.001) and 0.232 (χ^2^ = 180.67, *P* < 0.001). After the Delphi procedure, 37 items were included in Version 2.

Another six experts (two researchers with expertise in stroke patients, two researchers with expertise in scale development, one nursing expert working in the community, and one researcher with expertise in caregivers) were invited to assess content validity of Version 2. Item content validity index (I-CVI) and scale content validity index (S-CVI) were used to evaluate content validity, using a four-point scale ranging from 1 (not relevant) to 4 (highly relevant) (38). The I-CVI values ranged from 0.83 to 1.00, and the value of S-CVI was 0.97.

A pilot survey was conducted with 30 family caregivers. The survey took 5 to 8 min to complete and the items were clearly stated.

### Phase Three: Psychometric Evaluation of the Scale (from Version 2 to Version 3)

Construct validity was assessed by an exploratory factor analysis (EFA) to identify the possible components in the scale. Moreover, criterion-related validity, internal consistency reliability, and test-retest reliability were used to evaluate the validity and reliability. To perform EFA, the sample size must be greater than 300 (39). Ten percent of the family caregivers were randomly chosen again after 2 weeks to assess test-retest reliability (39). Version 2 was refined into Version 3 (26 items) after EFA. Then, confirmatory factor analysis (CFA) was performed to verify the components of Version 3. Additionally, convergent validity was used to evaluate validity. According to Boomsma’s advice, the minimal sample size for performing CFA is 300 (40).

### Measures

Participants’ demographic characteristics included gender, age, marital status, education, household income per month, health insurance, daily hours of caring, relationship with patients for family caregivers, and stroke patients’ severity of disability (Barthel Index) (35).

Version 2 of the Caregiver Benefit Finding Scale was used for the first survey in order to perform EFA. Version 3 was used for the second survey to perform CFA.

Positive Aspects of Caregiving (Chinese version; PAC-C) (22): PAC-C was used to evaluate the scale’s criterion-related validity, which is a nine-item self-report tool, including self-affirmation and outlook on life as two components. PAC-C items are scored from 1 (strongly disagree) to 5 (strongly agree) (22). PAC-C has an adequate internal consistency reliability, with the Cronbach’α of 0.90 (41).

### Data Analysis

Data were analyzed using IBM SPSS, Version 21.0 software and AMOS 17.0. The demographic characteristics of patients with stroke and their caregivers were analyzed descriptively and presented as numbers and percentages.

A Pearson correlation analysis was conducted to evaluate the item total correlation, and the value of item total correlation > 0.4 with a statistical significance testing was considered to indicate a desirable discriminating power and the criteria-related validity. Extreme group (27% and 73% of the score of the Caregiver Benefits Finding Scale) comparison was performed using an independent-samples *t*-test.

EFA was performed to explore construct validity under the situation that the Kaiser-Meyer-Olkin measure of sampling adequacy ≥ 0.8 and the Bartlett’s test of sphericity with *P* < 0.05 (42). Additional criteria included a factor loading of at least 0.4, with the difference between a loading and any cross loading of at least 0.15 for an item to remain on its factor. Each factor was also required to have at least three items for that factor to be retained.

CFA with the normal theory maximum likelihood estimation was then conducted to verify the construct validity (43). The value of relative chi-square (χ2/df) < 2, the values of goodness-of-fit index (GFI), the adjusted GFI (AGFI), a comparative fit index (CFI), and Bentler and Bonett’s normed-fit index (NFI) ≥ 0.9, and the value of a root mean square error of approximation (RMSEA) < 0.06 were considered to indicate an acceptable model fit (44). After conducting CFA, average variance extracted (AVE) and composite reliability were used to examine the convergent validity. The value of AVE > 0.5 and value of composite reliability > 0.7 were considered evidence of convergent validity.

Cronbach’s α and a model-based internal consistency index were used to evaluate the internal consistency reliability of the scale (45). A Pearson correlation analysis was used to assess the test-retest reliability, and the Guttman Split-half coefficient was used to evaluate the split-half reliability.

### Ethical Consideration

This study was approved by the Ethical Committee of Zhengzhou University. Informed consent statements were obtained from each participating hospital and community. All study participants agreed to join voluntarily and signed informed consent statements.

## Results

### Sample Characteristics

A total of 710 (20 for the semi-structured interview) family caregivers of stroke survivors were recruited at different phases. Of these, 340 participants were recruited to obtain data for EFA and 315 answered the questionnaires. Eight invalid questionnaires were removed. A total of 307 questionnaires were analyzed. Among those caregivers, 35 participants were randomly chosen to answer the questionnaires again 2 weeks later. Another 350 participants were recruited to obtain data for CFA, and 320 caregivers answered the questionnaires. Nine invalid questionnaires were removed and a total of 311 questionnaires were analyzed. The demographic characteristics of participants for recruited for EFA and CFA are listed in **Table 1**.

**Table 1:** Characteristics of the participants.

Variable	Variable category	EFA (n = 307)N (%)	CFA (n = 311)N (%)
**Age (years old)**	<45	110 (35.8)	151 (48.6)
	45~	140 (45.6)	100 (32.1)
	60~	57 (18.6)	60 (19.3)
**Gender**	Male	117 (38.1)	132 (42.4)
	Female	190 (61.9)	179 (57.6)
**Marital status**	Married	264 (86.0)	270 (86.8)
	Single/divorced/widowed	43 (14.0)	41 (13.2)
**Education level**	Primary	48 (15.6)	34 (10.9)
	Secondary	84 (27.4)	86 (27.7)
	High school	92 (30.0)	76 (24.4)
	University and above	83 (27.0)	115 (37.0)
**Household income per month (Yuan)**	<1000	36 (11.7)	30 (9.6)
	1000∼	45 (14.7)	44 (14.1)
	2000∼	99 (32.2)	67 (21.6)
	3000∼	127 (41.4)	170 (54.7)
**Relationship with the patients**	Spouse	110 (35.8)	100 (32.2)
	Daughters/sons	149 (48.5)	151 (48.5)
	Parents	38 (12.4)	36 (11.5)
	Other relatives	10 (3.3)	24 (7.8)
**Daily hours caring**	4~	59 (19.2)	66 (21.2)
	8~	59 (19.2)	72 (23.2)
	12h~	189 (61.6)	173 (55.6)
**Health insurance**	Province level	22 (7.2)	5 (1.6)
	City level	174 (56.7)	248 (79.7)
	New Rural Cooperative	57 (18.6)	41 (13.2)
	Others	54 (17.6)	17 (5.5)
**Severity of disability of care receiver**	Minimum dependence	151 (49.2)	142(45.7)
	Moderate dependence	62 (20.2)	55(17.7)
	Maximum dependence	94 (30.6)	114(36.6)

### Item Analysis and Extreme Group Comparison

All 37 item total correlation values ranged from 0.529 to 0.838 (all with *P* < 0.001) and show good inter-item associations. Extreme groups were divided into high- and low-score groups. All comparisons showed a significant difference (all with *P* < 0.001).

### Results of EFA

**Figure 2:**
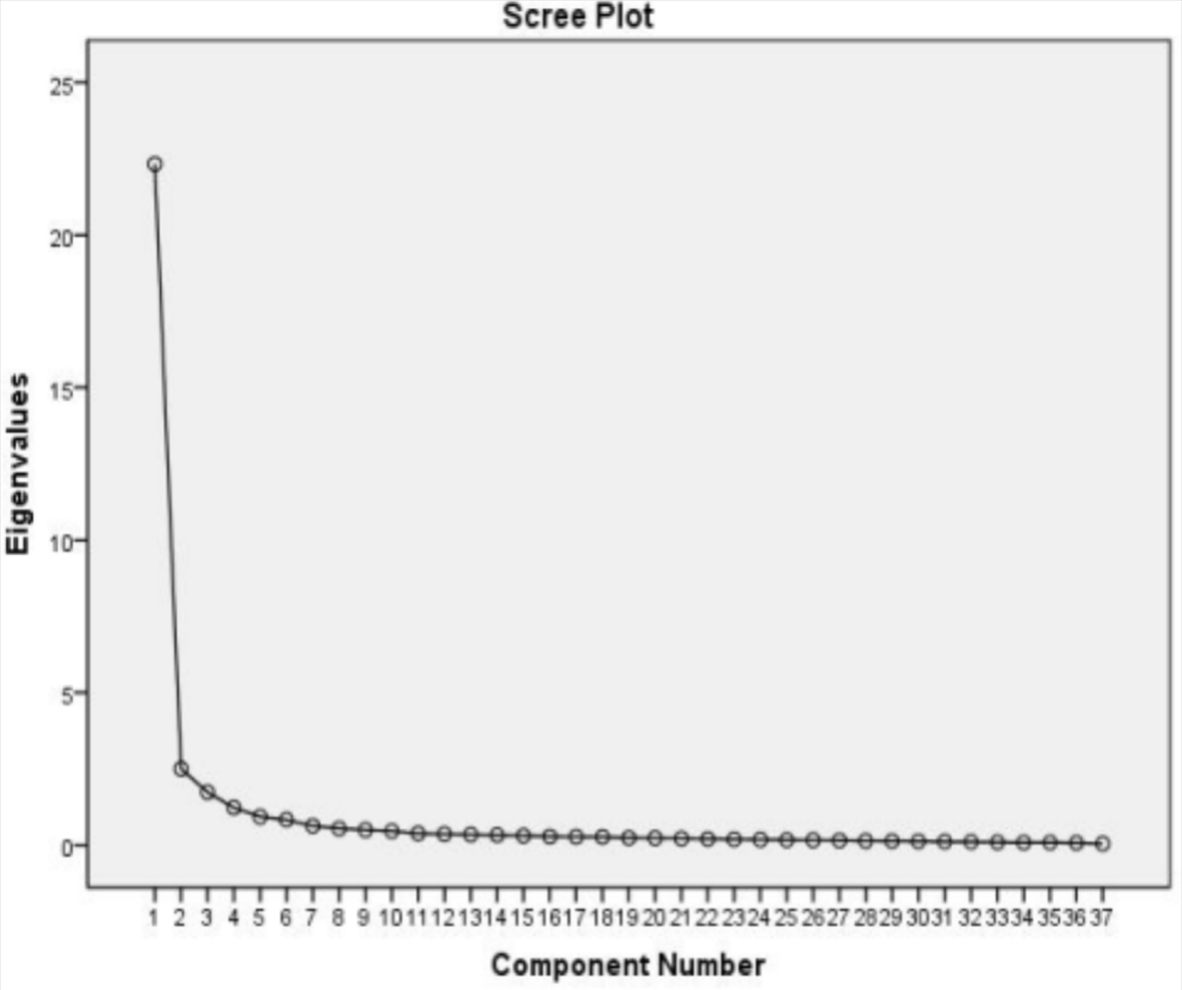
Scree plot for the exploratory factor analysis.

The Kaiser-Meyer-Olkin measure of sampling adequacy (0.965) and Bartlett’s test of sphericity (χ^2^ = 13330.00, *P* < 0.001) provided support for an EFA. Principal axis factoring with an oblimin rotation was chosen to perform the EFA, with the scree plot (**Figure 2**) used to determine the number of factors to rotate. Examination of the scree plot to determine the point at which the line/slope begins to flatten yielded four as the best starting point. Accordingly, solutions with three, four, and five factors were examined to find the most valid solution. As a result, 11 items were removed and EFA extracted four factors that accounted for 74.15% of the total variance with 26 items. Based on factor loadings, these four factors were termed “personal growth (seven items),” “health promotion (seven items),” “family growth (eight items),” and “self-sublimation (four items)” as shown in the component matrix (**Table 2**).

**Table 2:** Four factors extracted from factor analysis using matrix rotation (n = 307).

Taking care of patients				
	Factor 1	Factor 2	Factor 3	Factor 4
**Personal growth**				
Made me continuously improve my problem solving skills	0.911			
Made me improve my ability to care	0.861			
Made me cope with stress and difficulties better	0.818			
Made me know more about diseases	0.770			
Made me become more careful	0.749			
Made me improve my efficiency	0.688			
Made me improve my ability to guide others in healthy living	0.555			
**Health promotion**				
Made me pay more attention to the health of myself and other family members		0.816		
Made me focus on healthy eating		0.807		
Made me develop good habits		0.770		
Made me become aware of the significance of my health to the family and society		0.764		
Made me quit bad habits		0.716		
Made me see myself stronger and more brave		0.628		
Made me see things more positively		0.555		
**Family growth**				
Made our families become more united and harmonious			0.888	
Made me become more closer to my family			0.793	
Made my other family members have more time to do other things			0.672	
Made me feel more caring and support from my family			0.671	
Made me inherit and carry forward the tradition of loving each other			0.509	
Made my other family members become aware of love, giving and responsibility			0.502	
Made me spend more time with patients			0.500	
Made me be regarded as a good example by my family and friends			0.464	
**Self-sublimation**				
Made me feel more grateful and valued				0.781
Made me gain affirmation and praise				0.594
Made me feel more useful				0.558
Made me feel the sense of achievements				0.551

### Criterion-Related Validity

The Caregiver Benefit Finding Scale and PAC-C were in positively correlated (*r* = 0.760, *P* < 0.01), and all domains were also positively correlated (all *P* < 0.01).

### Reliability

The model-based internal consistency index of the scale was 0.845. The Cronbach’s α of each component was between 0.885 and 0.953. The values of split-half and test-retest reliability were 0.929 and 0.994 respectively.

### Results of CFA

The initial model had a χ^2^/df of 1.660 (*P* < 0.001), with GFI = 0.888, AGFI = 0.869, NFI = 0.932, IFI = 0.972, CFI = 0.972, and RMSEA = 0.046. The modification index indicated that the model fit could be improved. Ten correlation covariances were added, which could be explained in the content. Thus, the modified model produced a χ^2^/df of 1.254 (*P* = 0.002), with GFI = 0.921, AGFI = 0.901, NFI = 0.951, IFI = 0.990, CFI = 0.990, and RMSEA = 0.029. Although the χ^2^ is significant, this significance may exist because of the larger sample size. Overall, the model is a good fit and confirmed the results of EFA that suggested the Caregiver Benefit Finding Scale had four factors.

The value of AVE in the four domains ranged from 0.621 to 0.700. The value of composite reliability ranged from 0.891 to 0.942, which indicated a good convergent validity.

## Discussion

The main objectives of this study were to develop and analyze benefit finding psychometric properties and to develop a caregiver benefit finding scale to measure benefit finding for stroke family caregivers. The final scale comprises 26 items and possesses good validity and reliability. This finding expands the study of benefit finding by proposing a tool to directly measure benefit finding that can be used in the specific context of family caregiving of stroke survivors (see below section 4.1 for applications).

The EFA supported a four-dimensional scale structure, comprising a sense of personal growth, health promotion, family growth, and self-sublimation. Compared with the previous scales, the item “Made me be regarded as a good example by my family and friends” in the family growth domain was new added, and the health promotion and self-sublimation domains were new constructs. In Chinese culture, caregivers believe it is their duty and responsibility to care for their family members (32), and they are proud to be role models and set standards of care for the next generation (34); thus, they obviously perceive setting a good example for their families and friends as a benefit. Additionally, Chinese family caregivers place patients at the center of attention rather than themselves (32), and they are willing to do anything for their loved ones; thus, they promote healthier behaviors to take better care of stroke patients. Moreover, the Chinese government encourages the caregivers to take initiative in caring for their loved ones who need support at home (46), and thus caregivers possessed a great sense of contentment and value given recognition from their family and society. In addition, the CFA was also conducted to confirm the four-dimensional scale structure. The fit indices of CFA were satisfied. PAC-C was significantly correlated with the scale and all of its component factors, indicating that the Caregiver Benefit Finding Scale had acceptable criterion-related validity.

In this study, the “traditional” methods (Cronbach’s α) and an innovative method (i.e.,model-based internal consistency) were both used to test reliability (45). Cronbach’s α may not be the best method to test reliability of a multidimensional scale such as the Caregiver Benefit Finding Scale. As a result, the model-based internal consistency index for the total scale was 0.845. Cronbach’s α had a range of 0.885 to 0.953 for the subscales, which indicated that the scale had acceptable internal consistency. Moreover, split-half reliability was 0.929, and test-retest reliability was 0.994, which confirmed the stability of the tool.

The survey with the Caregiver Benefit Finding Scale was conducted with 350 family caregivers being surveyed. A total of 320 caregivers responded with nine of the surveys judged as invalid, an effective rate of 97.46% (47). It should be noted that it took caregivers 9 min to complete the survey which suggests this Caregiver Benefit Finding Scale has some advantages for the caregivers who are busy caring patients and with a limited amount of time (47). In addition, caregivers thought that the items were clear and easy to understand which confirms the idea that the effort expended in answering the survey was acceptable to caregivers and could be used further.

### Implications for Clinical and Research Practice

The Caregiver Benefit Finding Scale has important applications in the clinical and research areas. First, the scale provides a tool to measure the benefit finding of caregivers during acute and chronic phases of stroke recovery. Caregivers with a low level of benefit finding may be identified as a risk factor for negative outcome, requiring further assistance and resources (10). Second, the scale provides a tool to further study the mechanism of benefit finding and the experience of family caregivers, which includes both negative and positive experiences (48). Third, the scale provides a perspective on benefit finding intervention, which could focus on the personal growth, health promotion, family growth, and self-sublimation. Fourth, the Scale can be implemented as a measure in studies of interventions attempting to improve the mental health of family caregivers. Finally, the scale may help researchers to better understand how benefit finding may affect the outcomes of stroke patients (49).

### Limitations

The study has limitations. First, the participants in the interviews in phase 1 were self-selected and came from a relatively affluent region of China; thus, their experiences may not reflect those of other caregivers in less affluent or rural areas in China. Moreover, we selected caregivers for the interviews in phase 1 who were dealing with a wide duration of illness (4 months to 14 years) to identify various types of benefit finding in a qualitative way. However, we did not explore the differences in benefit finding as perceived by caregivers at different stages. It would be advisable to perform a longitudinal to examine the trajectories of the levels and different types of benefit finding. Second, all caregivers participating in this study were living in Zhengzhou. The fact that they all came from one particular geographical area may have a limited application. Thus, it would be advisable to conduct the study using a larger and more diverse sample from different areas in China. This expanded study would allow more confidence in using the Caregiver Benefit Finding Scale to confirm the psychometric properties.

## Conclusion

This study described the development of a Caregiver Benefit Finding Scale, a reliable and valid tool to evaluate important aspects of the experience of Chinese family caregivers taking care of stroke patients. The scale is shown to have adequate psychometric properties, which could be used to measure the positive experience that caregivers might have as a direct result of being family caregivers for stroke patients.

## Data Availability Statement

The raw data supporting the conclusions of this article will be made available by the authors, without undue reservation.

## Ethics Statement

The studies involving human participants were reviewed and approved by ethical committee of Zhengzhou University. The patients/participants provided their written informed consent to participate in this study.

## Author Contributions

Y-XM was involved in design, data collection, analysis, and writing. B-LL was involved in data collection and analysis. W-HZ was involved in design and writing. S-SW was involved in data collection and writing. Z-XZ, D-BY, and DC were involved in design and writing. All authors contributed to the article and approved the submitted version.

## Funding

This study was funded by the China Postdoctoral Science Foundation (funding numbers:2019M652589); the Medical Science and Technology Breakthrough Plan Project of Henan province (funding number:SBGJ2018052); the National Natural Science Foundation of China (funding numbers: U1404814); the Henan Province Science Technology Innovation Talent Support Plan (funding numbers: 17HASTIT048); and the Science and Technology Department of Henan Province (funding number 134200510018). The funders had no role in the study design, data collection and analysis, decision to publish, or preparation of the manuscript.

## Conflict of Interest

The authors declare that the research was conducted in the absence of any commercial or financial relationships that could be construed as a potential conflict of interest.
