# Supplementary material for: Creating a Caregiver Benefit Finding Scale of Family Caregivers of Stroke Survivors: Development and Psychometric Evaluation
Source: Front Psychiatry. 2020 Jul 29;11:734. doi: 10.3389/fpsyt.2020.00734 (PMC7403500; doi:10.3389/fpsyt.2020.00734)
Supplement: Supplementary file 1 [file Table_1.doc]

Table 1S Previous scales related to caregiving benefits findings

| **NO.** | **Scale Name** | **Developers** | **Factor number** | **Testing sample** | **Item**  **Number** | **Cronbach’s alphas** | **Rating** |
| --- | --- | --- | --- | --- | --- | --- | --- |
| 1 | Stress Related Growth Scale | Park, Cohen, Murch,1996 | One/three/seven factors (not sure) | College students | 50/43/15 | 0.97 | 3-point scale |
| 2 | Post Traumatic Growth Inventory | Tedeschi  & Calhoun,  1996 | Five factors  (new possibilities, relating to others, personal strength, spiritual change,  appreciation of life) | Persons who have experienced  traumatic events | 21 | 0.87 | 6-point scale |
| 3 | Positive Aspects of Caregiving | Tarlow et al.,  2004 | Two factors (self-affirmation and outlook-on-life) | Caregivers of dementia patients | 9 | 0.89 | 5-point scale |
| 4 | Gains in Alzheimer’s Care Instrument | Yap et al,  2010 | Three factors  ( personal growth, gains in relationships,  higher level gains) | Caregivers of dementia patients | 10 | 0.89 | 4-point scale |
| 5 | Reward of Caregiving Scale | Archbold,  1993 | Three/ two factors | Caregivers of dementia patients/ palliative patients | 15/10 | 0.94 | 5-point scale |
| 6 | Scale for Positive Aspects of Caregiving Experience | Kate et al,  2012 | Four factors  (caregiving personal gains, motivation for caregiving role, caregiver satisfaction, self-esteem, social aspects of caring) | Caregivers of  patients with severe mental disorders | 44 | 0.923 | 5-point scale |
| 7 | Benefit Finding Scale | Antoni et al,  2001 | One factor | Patients with breast cancer | 17 | 0.95 | 5-point scale |
| 8 | Benefit Finding in Multiple Sclerosis scale | Pakenham et al, 2008 | Six factors  (enriched relationship, spiritual growth, family relations growth, life-style gains, inspiration, relationship opportunities) | Caregivers of those with multiple sclerosis | 27 | 0.92 | 3-point scale |
| 9 | Gratifications of Caregiving Questionnaire | Greenberg et al., 1994 | One factor | Parents of mentally ill adult  Children | 8 | 0.86 | 5-point scale |
| 10 | Caregiver Benefit Index | Meltzer et al,  2001 | - | Caregivers of liver and lung transplant candidates | 5 | - | Yes/no |
